# A solution-processed quaternary oxide system obtained at low-temperature using a vertical diffusion technique

**DOI:** 10.1038/srep43216

**Published:** 2017-02-23

**Authors:** Seokhyun Yoon, Si Joon Kim, Young Jun Tak, Hyun Jae Kim

**Affiliations:** 1 School of Electrical and Electronic Engineering, Yonsei University, 50 Yonsei-ro, Seodaemun-gu, Seoul 120-749, Republic of Korea

## Abstract

We report a method for fabricating solution-processed quaternary In-Ga-Zn-O (IGZO) thin-film transistors (TFTs) at low annealing temperatures using a vertical diffusion technique (VDT). The VDT is a deposition process for spin-coating binary and ternary oxide layers consecutively and annealing at once. With the VDT, uniform and dense quaternary oxide layers were fabricated at lower temperatures (280 °C). Compared to conventional IGZO and ternary In-Zn-O (IZO) thin films, VDT IGZO thin film had higher density of the metal-oxide bonds and lower density of the oxygen vacancies. The field-effect mobility of VDT IGZO TFT increased three times with an improved stability under positive bias stress than IZO TFT due to the reduction in oxygen vacancies. Therefore, the VDT process is a simple method that reduces the processing temperature without any additional treatment for quaternary oxide semiconductors with uniform layers.

Silicon-based thin-film transistors (TFTs) have been used for backplanes since the birth of active-matrix liquid-crystal displays and active-matrix organic light-emitting diode (AMOLED) displays^1^,^2^. However, amorphous Si TFTs have poor electrical performance, and low-temperature polycrystalline Si TFTs have low scalability and high fabrication costs despite their enhanced electrical performance. Because it is difficult to apply Si-based TFTs to large high-resolution displays, many researchers have focused on modifying Si-based TFTs. Among the alternatives, there has been extensive research of oxide TFTs due to their high mobility, low off-current, high transparency, high uniformity, and simple deposition methods^3^,^4^,^5^,^6^,^7^,^8^,^9^. Some companies have already produced commercial products, including large AMOLED televisions, smart phones, and tablets.

To maximize price competitiveness and enhance productivity, a solution process is necessary for oxide semiconductors^8^,^9^. However, solution-processed oxide TFTs have an inherent problem: poor electrical performance compared with vacuum-processed oxide TFTs. Higher processing temperatures are generally required to overcome this problem. However, at higher processing temperatures, it can be difficult to produce flexible devices because the maximum processing temperature of flexible substrates is below 300 °C. To resolve this issue, many researchers have focused on lowering the processing temperature while maintaining high electrical performance. There are three ways to lower the processing temperature: solution modulation, additional treatment, and structure modulation. For solution modulation, some research groups have tried different precursors^10^,^11^ and solvents^12^,^13^,^14^. The processing temperature can be reduced by doping atoms^15^ and various additives^16^,^17^ introduced to oxide semiconductors. As additional treatments, high-pressure annealing (HPA)^18^,^19^,^20^, vacuum annealing^14^,^21^,^22^, ultraviolet (UV) annealing^21^,^23^, O_2_/O_3_ annealing^24^, and microwave annealing^25^,^26^ have been examined. Lastly, by changing or modulating the gate insulator materials^27^,^28^,^29^ and adopting a dual-channel layer^30^, the processing temperature can be reduced. However, in previous studies, mostly binary (In-O) or ternary (In-Zn-O (IZO)) oxides were used, because these have lower processing temperatures compared with quaternary oxides, which also require an additional process with special equipment. For electrical stability, it is necessary to use a quaternary oxide (In-Ga-Zn-O (IGZO)) with a carrier suppressor (Ga), as these are the only oxide semiconductors used in commercial products.

In this study, we introduce a simple method to reduce the processing temperature for a quaternary oxide: the vertical diffusion technique (VDT). The VDT is a process used to deposit two oxide layers successively and anneal them simultaneously in order to effectively facilitate diffusion between each layer. With the VDT, uniform IGZO TFTs were fabricated with lower processing temperatures and superior electrical performance, without any additional treatment. The VDT enables a significant reduction in processing temperatures to below 300 °C, while maintaining the electrical performance of the IGZO TFTs. Thus, this approach is expected to be useful in the fabrication of flexible oxide TFTs due to lower fabrication cost and simple process compared to aforementioned methods.

## Experimental Procedure

### Materials

Indium nitrate hydrate [In(NO_3_)_3_∙xH_2_O], gallium nitrate hydrate [Ga(NO_3_)_3_∙xH_2_O], and zinc nitrate hydrate [Zn(NO_3_)_2_∙xH_2_O] precursors were used for the IGZO solution. The precursors were dissolved in 2-methoxyethanol [CH_3_OC_2_H_4_OH]. To enhance the electrical properties, nitric acid (HNO_3_) was added to the solution. All chemicals were purchased from Sigma-Aldrich and used without further purification.

### Solution preparation

IZO, GaO, and IGZO solutions were prepared. We controlled the molarity of each solution to achieve the desired total atomic composition. The mole ratios were 5:2:1 = In:Ga:Zn for IGZO and 5:1 = In:Zn for IZO. The molarities of the IGZO, IZO, and GaO solutions were 0.4, 0.3, and 0.1 M, respectively. The total atomic composition (In, Ga, and Zn) was the same in the IGZO and VDT IGZO thin films. The solutions were stirred at 60 °C for 1 h, and the precursors dissolved entirely. The solutions were filtered through a Whatman 0.2-μm polytetrafluoroethylene (PTFE) syringe filter, and aged for at least than 24 h in ambient air.

### TFT fabrication

The VDT TFTs exhibited an inverted staggered structure. The substrate was 120-nm-thick SiO_2_ thermally oxidized on heavily p-doped Si. The solutions were spin-coated on the substrate. In this experiment, samples of IGZO, IZO, and VDT IGZO were prepared. The conventional IGZO and IZO thin films were pre-annealed for 5 min at 100 °C. For the VDT IGZO, GaO was first spin-coated and pre-annealed for 5 min at 100 °C. Then, IZO was spin-coated on the GaO-coated thin film, and pre-annealed for 5 min at 100 °C. After pre-annealing, all of the samples were post-annealed 280 °C for 4 h. Aluminum (Al) was used for the source and drain electrodes and was deposited on the IGZO thin film via a shadow mask by thermal evaporation. The channel length and width of the IGZO TFTs were 150 μm and 1000 μm, respectively. The HPA process was conducted under 1 MPa O_2_ at 280 °C for 4 h, as a post-annealing process.

### Characterization

An electrical measurement system with a probe station and a semiconductor parameter analyzer (HP 4156 C) was used to measure the transfer characteristics when V_DS_ was 30 V and V_GS_ was swept from −30 V to + 30 V. For positive bias stress (PBS), stress conditions were applied (V_GS_ = 20 V) for 1000 s. Depth-X-ray photoelectron spectroscopy (XPS) and time-of-flight secondary ion mass spectrometry (TOF-SIMS) were conducted using a TOF-SIMS 5 system (iONTOF, Germany), equipped with a Cs gun (Thermo Scientific, U.K.) operating at 1 keV with a monochromatic Al X-ray source (Al Kα line: 1486.6 eV). All of the XPS peaks were calibrated using the C 1 s peak, centered at 284.8 eV. AFM analysis (JPK instrument, Germany) was performed to measure RMS (root mean square) and peak-to-valley roughness.

## Results and Discussion

**Figure 1:**
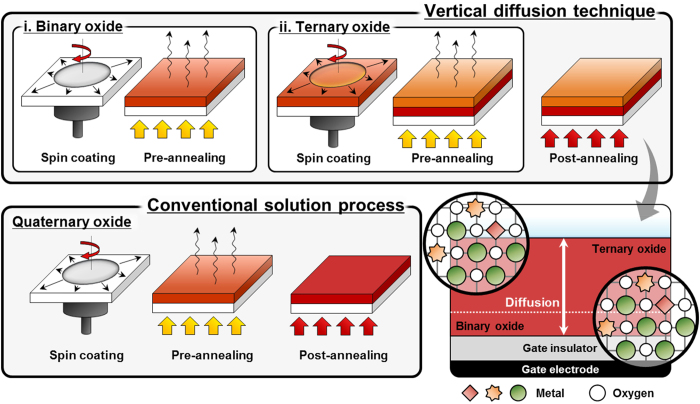
Schematic diagram of VDT and conventional processes for quaternary oxide.

Figure 1 shows a schematic diagram of the experimental process for conventional and vertically diffused IGZO thin films. Because the optimized molar ratio of IGZO is In:Ga:Zn = 5:2:1, we used 0.4 M IGZO with In:Ga:Zn = 5:2:1 for the reference^31^, and 0.3 M IZO with In:Zn = 5:1 and 0.1 M GaO were prepared for the VDT. For the VDT, we spin-coated the substrate with GaO and IZO; each layer was pre-annealed. The IZO/GaO thin film, i.e., the VDT IGZO, was post-annealed at 280 °C.

**Figure 2:**
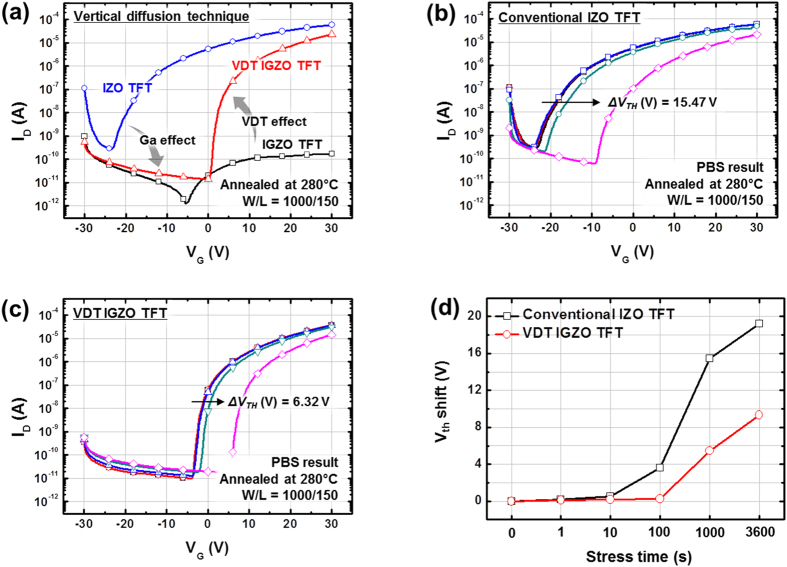
(**a**) Transfer characteristics of conventional IGZO, conventional IZO, and VDT IGZO TFTs; PBS results of (**b**) conventional IZO and (**c**) VDT IGZO TFTs. (**d**) variation of V_TH_ shift under PBS for conventional IZO and VDT IGZO TFTs according to stress time.

Figure 2(a) shows the transfer curves of the IGZO, IZO, and VDT IGZO TFTs; Table 1 summarizes their electrical properties, including the field-effect mobility (μ_FET_), maximum on-current/minimum off-current (on/off ratio), subthreshold swing (S.S), equivalent maximum density of states between channel and gate insulator (N_max_), threshold voltage (V_TH_), and on-current maximum (I_on,max_). The IGZO TFTs deposited using the conventional method did not have suitable transfer characteristics at 280 °C because 280 °C is insufficient to form an IGZO thin film. In contrast, the IZO and VDT IGZO TFTs had suitable transfer characteristics. Generally, the processing temperature of ternary oxide thin film is lower than that of a quaternary oxide thin film (IGZO) and IZO is the ternary oxide most commonly used for lower-temperature processes. For this reason, IZO TFTs showed appropriate transfer curves at 280 °C. The VDT IGZO TFTs also had suitable transfer characteristics despite being a quaternary oxide. With the GaO layer, the μ_FET_ improved from 0.40 to 1.26 cm^2^/Vs without decreasing I_on,max_. Moreover, SS decreased from 1.92 to 1.16 V/dec. with the GaO layer. Therefore, Ga from the GaO layer successfully controlled the carrier concentration in the IZO thin film as a carrier suppressor by reducing oxygen vacancies in the oxide thin film^6^,^8^,^9^,^32^,^33^. Consequently, the mobility increased by 215% and the S.S improved by 40%. Moreover, as shown in Fig. 2(b) and (c), the V_TH_ shift under PBS for 1000 s also improved from 15.47 to 6.32 V (by 59%) with the VDT process and Fig. 2(d) shows variation of V_TH_ shift under PBS for 3600 s. This result was correlated with the reduced N_max_ and reduction in oxygen vacancies, as oxygen vacancies give rise to PBS instability^34^,^35^,^36^.

**Figure 3:**
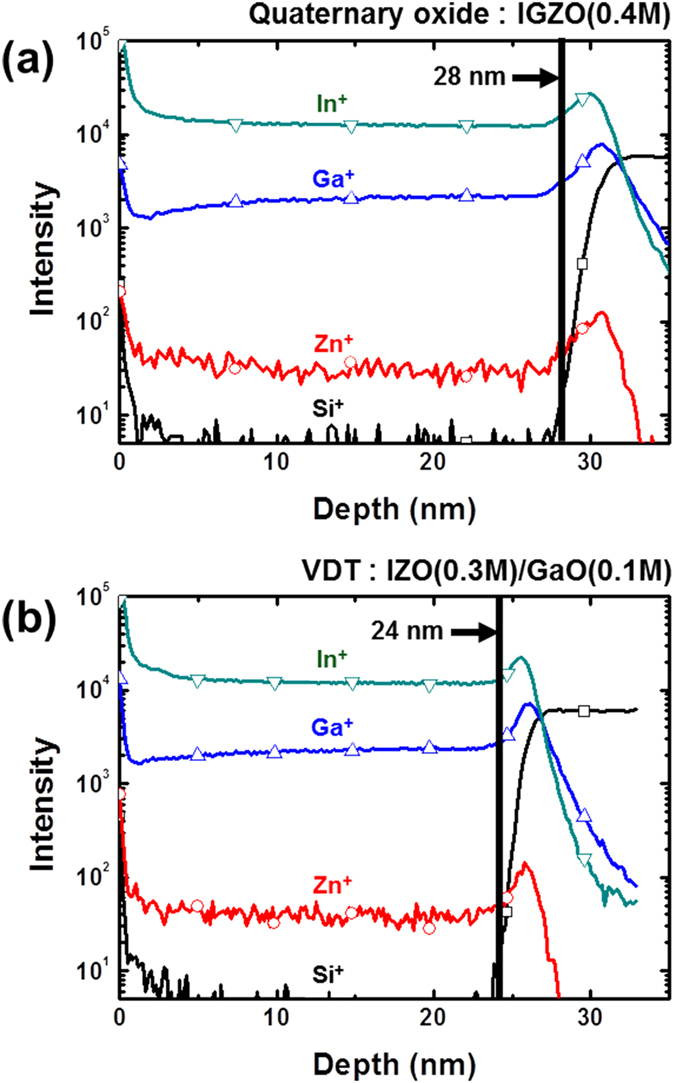
TOF-SIMS analysis of (**a**) conventional IGZO and (**b**) VDT IGZO thin-films.

**Figure 4:**
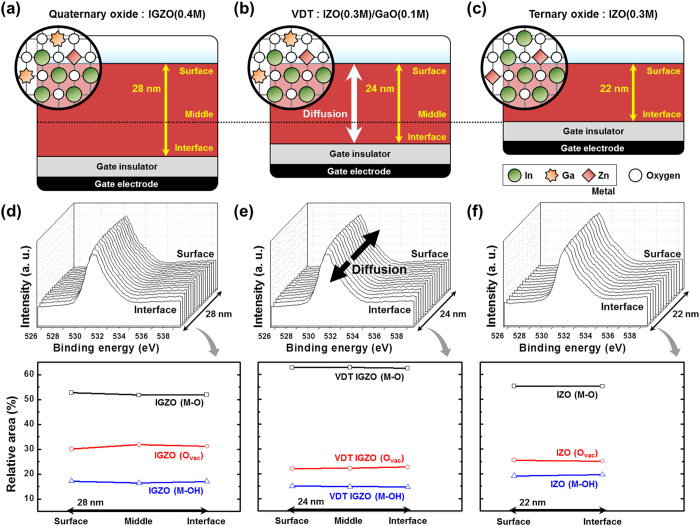
The thickness of metal-oxide semiconductor layers calculated by spectroscopic ellipsometry; (**a**) 28-nm-thick IGZO, (**b**) 24-nm-thick IZO/GaO, and (**c**) 22-nm-thick IZO. O 1 s peak and its variation of XPS data according to the depth of (**d**) IGZO, (**e**) IZO/GaO, and (f) IZO.

To confirm the thickness and atomic composition, TOF-SIMS was performed for the conventional IGZO and VDT IGZO thin films, and the thickness of the samples was calculated by spectroscopic ellipsometry, as shown in Figs 3 and 4(a–c). Both IGZO and VDT IGZO had uniform atomic ratios with respect to depth, although some irregular peaks were observed at the interface due to matrix effects^37^,^38^. Although the first (GaO) and second (IZO) oxide layers were deposited separately, the atoms uniformly diffused into the thin film during the post-annealing process. Moreover, no matrix effects were observed in the VDT IGZO layer, which means that there was no interface in the thin film. In addition, densification occurred during the VDT process because the VDT IGZO was thinner than IGZO. Therefore, the VDT process resulted in a uniform, dense thin film without an interface. Furthermore, to investigate densification effect for VDT process, RMS roughness was measured for conventional IGZO and VDT IGZO thin films using AFM analysis. Figure S1 show the results of AFM analysis for conventional IGZO and VDT IGZO thin films. As a result, the RMS and peak-to-valley roughness of conventional IGZO and VDT IGZO thin films were 0.348 nm and 0.279 nm, and 12.99 nm and 3.91 nm, respectively. Therefore, it should be noted that VDT process helps to recover pore sites caused by solvent evaporation resulting in densification of thin films.

Figure 4(d–f) shows the O 1 s XPS peaks according to the depth of the conventional IGZO, IZO, and VDT IGZO thin films. The O 1 s peaks did not change significantly with the depth of the oxide thin films. First, to confirm uniformity, we compared the O 1 s peaks of the three samples at the surface and interface between the channel and gate insulator layer. If the In, Ga, and Zn did not diffuse entirely in the VDT IGZO thin films, there would be different O 1 s peaks in the middle and at the IZO interface, because the initial VDT IGZO layers were IZO (from the middle to the surface) and GaO (from the interface to the middle). Therefore, to confirm diffusion, we analyzed the middle of the IGZO, which is the entirely diffused layer, and VDT IGZO.

All three O 1 s peaks were deconvoluted and centered at 530, 531, and 532 ± 0.5 eV; the three peaks corresponded to lower (metal oxide bonds), intermediate (oxygen vacancy), and higher (metal hydroxide species) binding energies, and the relative areas of the three peaks corresponded to M-O, O_vac_, and M-OH, respectively^39^,^40^. Figure 4(d–f) also shows the variation in the O 1 s peaks with respect to the depth of the IGZO, IZO, and VDT IGZO thin films. The variation in all of the O 1 s peaks (M-O, O_vac_, and M-OH) for the three samples was less than 1%, indicating that IGZO, IZO, and VDT IGZO had uniform M-O, O_vac_, and M-OH distributions from the surface to the interface, and all of the atoms in VDT IGZO diffused uniformly. These results concur with the previous results.

Figure S2 shows the O 1 s peaks at the surface of the IGZO, IZO, and VDT IGZO, and Figs S3 and S4 show the O 1 s peaks in the middle and at the interface of the three samples. At the surface, the M-Os, O_vac_s, and M-OHs of IGZO, IZO, and VDT IGZO were 52.78, 55.31, and 62.85%, 30.07, 25.51, and 22.09%, and 17.15, 19.17, and 15.06%, respectively. Because the IGZO thin film was not completely formed at 280 °C, the M-O of IGZO had the lowest value among the three samples. Compared with IZO, VDT IGZO had a higher M-O, indicating that the VDT process enabled a thin film to form at low temperature. Moreover, the VDT IGZO had lower O_vac_ due to diffusion of Ga, which is a carrier suppressor, in the IZO. Generally, the instability origin under PBS was electron trap sites; i.e., oxygen vacancies in the oxide semiconductor^34^,^35^,^36^. Due to the Ga effect, the oxygen vacancy and Nmax decreased, leading to improved PBS results for VDT IGZO.

**Figure 5:**
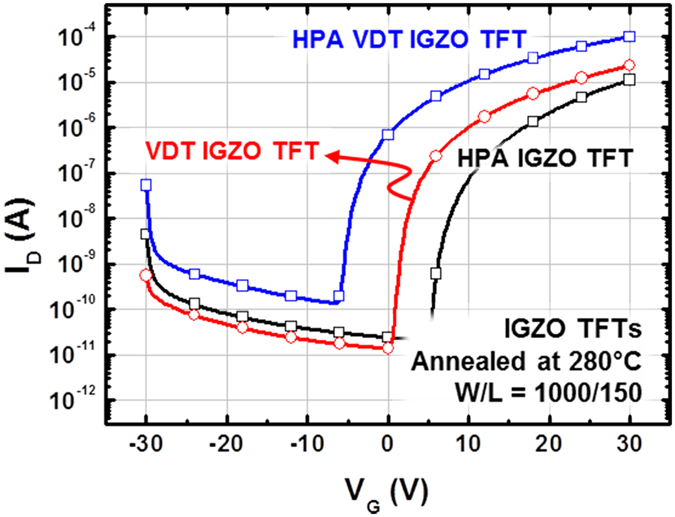
Transfer characteristics of HPA IGZO, VDT IGZO, and HPA VDT IGZO TFTs.

To enhance the electrical properties, the IGZO and VDT IGZO TFTs were subjected to HPA on flexible substrates as a post-treatment under 1 MPa O_2_. HPA effectively reduced the processing temperature and improved electrical performance, not only μ_FET_ but also the electrical stability^18^,^19^,^20^. As the IZO TFTs were inferior to VDT IGZO TFTs, the IGZO and VDT IGZO TFTs were used in the experiments, with IGZO TFTs serving as a reference. Figure 5 shows the transfer curves for the HPA IGZO, VDT IGZO, and HPA VDT IGZO TFTs; Table 2 summarizes the electrical parameters. With HPA, the IGZO TFTs had suitable transfer characteristics at 280 °C, as shown in Fig. 5. Although the IGZO TFTs were subject to HPA, which requires additional equipment, the VDT IGZO TFTs had superior electrical performance, in particular, a three-fold difference in μ_FET_. Moreover, HPA also improved the electrical performance of VDT IGZO TFTs. The μ_FET_ of the HPA VDT IGZO was 1.38 cm^2^/V·s, an improvement of 8.7%. Therefore, the VDT is a simple method that not only decreases the processing temperature but also improves the electrical properties; moreover, the VDT combined with HPA maximized the improvement in electrical performance.

## Conclusion

In this paper, we suggest a strategy to reduce the processing temperature for IGZO TFTs using vertical diffusion. With the VDT, uniform quaternary oxide layers were fabricated at lower temperatures, and VDT resulted in quaternary oxide TFTs with suitable transfer characteristics at 280 °C, without the need for additional treatment. It can be explained that VDT process enables to form high quality quaternary oxide film by diffusing atoms between gel-state binary and ternary oxide system. Therefore, by post-annealed at once, atoms of binary and ternary oxide are effectively diffused each layer resulting in formation of quaternary oxide film at low temperature. In contrast, conventional IGZO TFTs did not show proper transfer characteristics at 280 ^o^C because four kinds of atoms are difficult to make high metal-oxide-metal framework at this low temperature. The VDT IGZO TFTs had a higher μ_FET_ with a lower S.S than IZO TFTs due to the reduction in oxygen vacancies. The PBS results for the VDT IGZO TFTs were also superior to those of the IZO TFTs due to the Ga component, which is a carrier suppressor. Moreover, HPA improved the μ_FET_ of VDT IGZO TFTs by 8.7%, and the μ_FET_ of VDT IGZO increased three times than HPA IGZO. Therefore, the VDT process is a simple method that reduces the processing temperature without any additional treatment for quaternary oxide semiconductors with uniform layers.

## Additional Information

**How to cite this article:** Yoon, S. *et al*. A solution-processed quaternary oxide system obtained at low-temperature using a vertical diffusion technique. *Sci. Rep.* **7**, 43216; doi: 10.1038/srep43216 (2017).

**Publisher's note:** Springer Nature remains neutral with regard to jurisdictional claims in published maps and institutional affiliations.

## Supplementary Material

Supplementary Information

## Figures and Tables

**Table 1:** Electrical parameters of conventional IGZO, conventional IZO, and VDT IGZO TFTs annealed at 280 ^o^C.

Parameters	IGZO	IZO	VDT IGZO
*μ*_*FET*_ (cm^2^/Vs)	—	0.40	1.26
*On/off ratio*	—	2.11 × 10^5^	3.08 × 10^6^
*S.S* (V/decade)	—	1.92	1.16
*N*_*max*_ (/cm^2^)	—	5.61 × 10^12^	3.16 × 10^12^
*V*_*th*_ (V)	—	−10.17	−2.36
*I*_*on,max*_ (A)	—	5.77 × 10^−5^	2.30 × 10^5^

**Table 2:** Electrical parameters of conventional IGZO, HPA conventional IGZO, VDT IGZO and HPA VDT IGZO TFTs annealed at 280 ^o^C.

Parameters	HPA IGZO	VDT IGZO	HPA VDT IGZO
*μ*_*FET*_ (cm^2^/Vs)	0.41	1.26	1.38
*On/off ratio*	5.16 × 10^5^	3.08 × 10^6^	7.16 × 10^5^
*S.S* (V/decade)	0.86	1.16	0.90
*N*_*max*_ (/cm^2^)	2.43 × 10^12^	3.16 × 10^12^	2.54 × 10^12^
*V*_*th*_ (V)	13.34	−2.36	2.45
