# Supplementary material for: A solution-processed quaternary oxide system obtained at low-temperature using a vertical diffusion technique
Source: Sci Rep. 2017 Feb 23;7:43216. doi: 10.1038/srep43216 (PMC5322350; doi:10.1038/srep43216)
Supplement: Supplementary Information [file srep43216-s1.pdf]

# Supplementary Information for

## A solution-processed quaternary oxide system obtained at low-temperature using a vertical diffusion technique

*Seokhyun Yoon[1,2], Si Joon Kim[1,3], Young Jun Tak[1], and Hyun Jae Kim[1,★]*

[1]*School of Electrical and Electronic Engineering, Yonsei University, 50 Yonsei-ro, Seodaemun-gu, Seoul 120-749, Republic of Korea*

[2]*Samsung Display Co., Ltd., #181, Samsung-ro, Tangjeong-Myeon, Asan-City, Chungcheongnam-Do 336-741, Republic of Korea*

[3]*Department of Materials Science and Engineering, The University of Texas at Dallas, 800 W. Campbell Road, Richardson, Texas 75080-3021, United States*

[‡]S. Yoon, S. J. Kim, and Y. J. Tak contributed equally to this work.

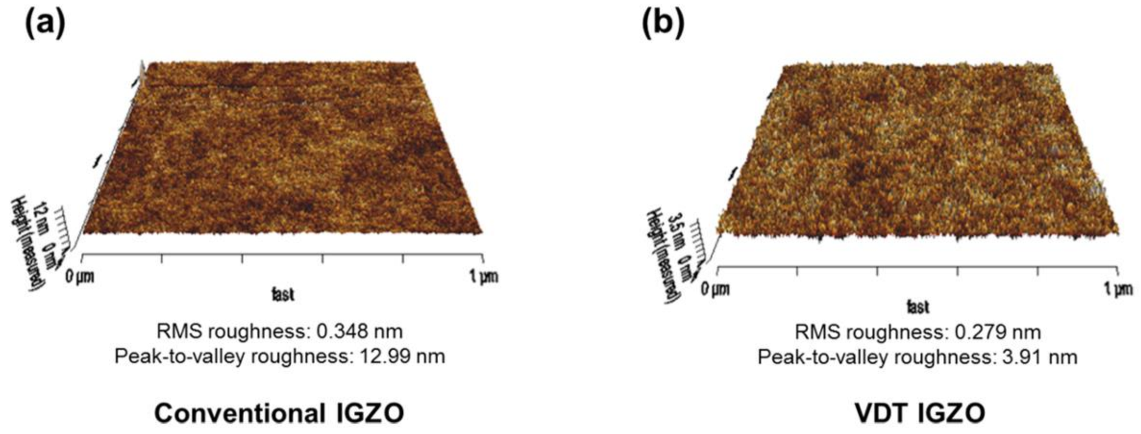


**Figure S1.** AFM images of (a) conventional IGZO and (b) VDT IGZO.

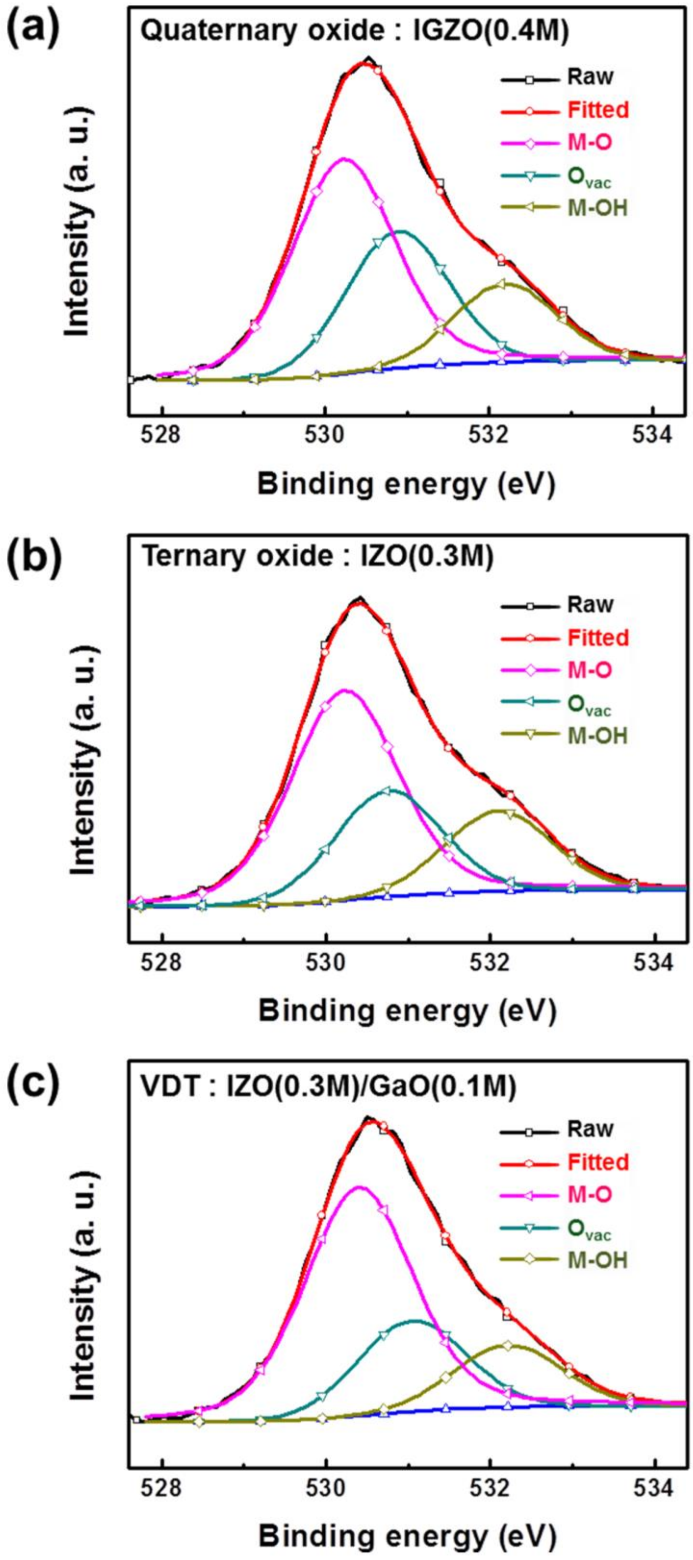


**Figure S2.** O 1s peak of XPS data at the surface of (d) IGZO, (e) IZO, and (f) IZO/GaO.

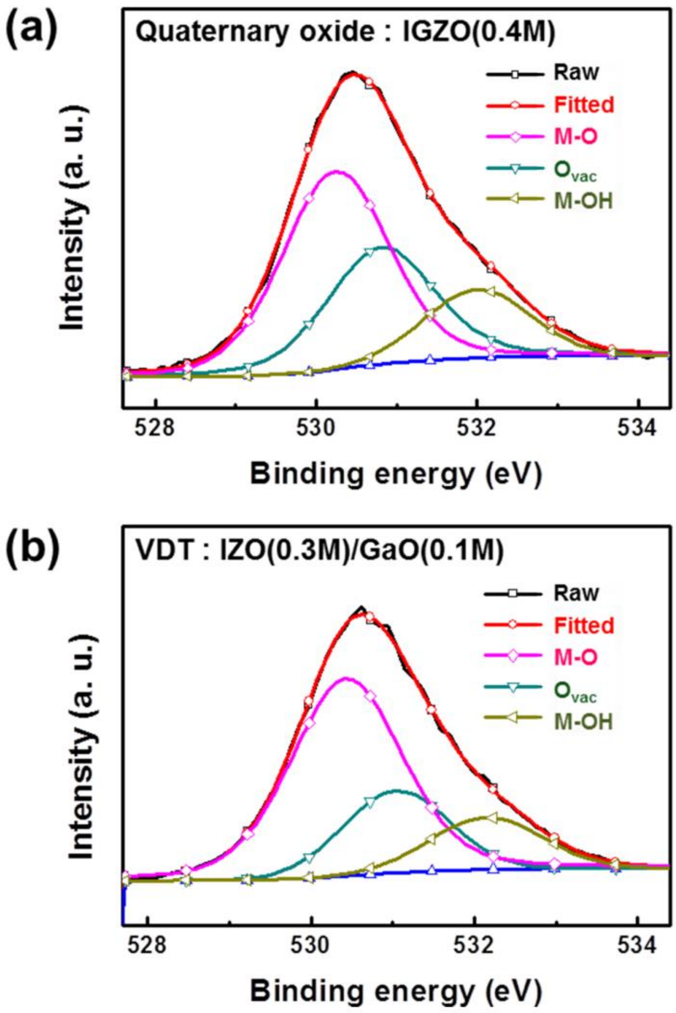


**Figure S3.** O 1s peak of XPS data at the middle of (a) IGZO and (b) VDT IGZO.

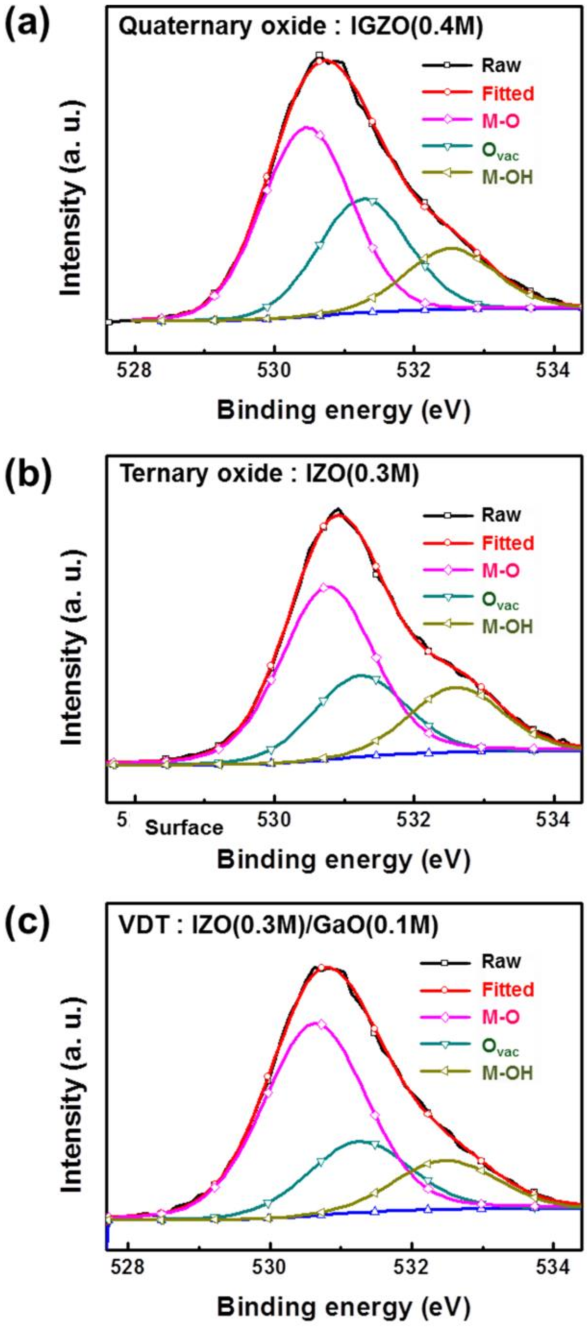


**Figure S4.** O 1s peak variation of XPS data at the interface according to depth of (a) IGZO and (b) IZO, and (c) VDT IGZO.
